# GMP-conformant on-site manufacturing of a CD133^+^ stem cell product for cardiovascular regeneration

**DOI:** 10.1186/s13287-016-0467-0

**Published:** 2017-02-10

**Authors:** Anna Skorska, Paula Müller, Ralf Gaebel, Jana Große, Heiko Lemcke, Cornelia A. Lux, Manuela Bastian, Frauke Hausburg, Nicole Zarniko, Sandra Bubritzki, Ulrike Ruch, Gudrun Tiedemann, Robert David, Gustav Steinhoff

**Affiliations:** 1 Reference and Translation Center for Cardiac Stem Cell Therapy (RTC), Department of Cardiac Surgery, Rostock University Medical Center, Schillingallee 68, Rostock, 18057 Germany; 2 Department Life, Light and Matter of the Interdisciplinary Faculty at Rostock University, Albert-Einstein Straße 25, Rostock, 18059 Germany; 3 Institute for Clinical Chemistry and Laboratory Medicine (ILAB), Rostock University Medical Center, Ernst-Heydemann-Straße 6, Rostock, 18057 Germany; 4 Department of Cardiac Surgery, Rostock University Medical Center, Schillingallee 35, Rostock, 18057 Germany

**Keywords:** Cardiovascular regeneration, Adult hematopoietic stem cells, Prodigy, Good Manufacturing Practice (GMP), Clinical translation, Advanced therapy medicinal product (ATMP), CD133^+^ cells, Stem cell transplantation

## Abstract

**Background:**

CD133^+^ stem cells represent a promising subpopulation for innovative cell-based therapies in cardiovascular regeneration. Several clinical trials have shown remarkable beneficial effects following their intramyocardial transplantation. Yet, the purification of CD133^+^ stem cells is typically performed in centralized clean room facilities using semi-automatic manufacturing processes based on magnetic cell sorting (MACS®). However, this requires time-consuming and cost-intensive logistics.

**Methods:**

CD133^+^ stem cells were purified from patient-derived sternal bone marrow using the recently developed automatic CliniMACS Prodigy® BM-133 System (Prodigy). The entire manufacturing process, as well as the subsequent quality control of the final cell product (CP), were realized on-site and in compliance with EU guidelines for Good Manufacturing Practice. The biological activity of automatically isolated CD133^+^ cells was evaluated and compared to manually isolated CD133^+^ cells via functional assays as well as immunofluorescence microscopy. In addition, the regenerative potential of purified stem cells was assessed 3 weeks after transplantation in immunodeficient mice which had been subjected to experimental myocardial infarction.

**Results:**

We established for the first time an on-site manufacturing procedure for stem CPs intended for the treatment of ischemic heart diseases using an automatized system. On average, 0.88 × 10^6^ viable CD133^+^ cells with a mean log_10_ depletion of 3.23 ± 0.19 of non-target cells were isolated. Furthermore, we demonstrated that these automatically isolated cells bear proliferation and differentiation capacities comparable to manually isolated cells in vitro. Moreover, the automatically generated CP shows equal cardiac regeneration potential in vivo.

**Conclusions:**

Our results indicate that the Prodigy is a powerful system for automatic manufacturing of a CD133^+^ CP within few hours. Compared to conventional manufacturing processes, future clinical application of this system offers multiple benefits including stable CP quality and on-site purification under reduced clean room requirements. This will allow saving of time, reduced logistics and diminished costs.

**Electronic supplementary material:**

The online version of this article (doi:10.1186/s13287-016-0467-0) contains supplementary material, which is available to authorized users.

## Background

Adult stem cells remain of particular interest as therapeutic agents for cardiac regeneration in ischemic heart disease [1–4]. Recently, several clinical trials showed an increased left ventricular ejection fraction (LVEF) and an improved regional perfusion following intramyocardial application of cluster of differentiation (CD) 133^+^ stem cells [5–9]. These adult cells represent a subset of CD34^+^ progenitors and are easily accessible from patient-derived bone marrow (BM) posing little ethical conflicts [10–12]. The regeneration potential of these cells is mainly based on their direct contribution to neovascularization and on their secretion of various paracrine factors activating pro-angiogenic mechanisms [13–16].

According to the European Medicines Agency (EMA) BM-derived CD133^+^ stem cells intended for the regeneration of human ischemic heart tissue are classified as an advanced therapy medicinal product (ATMP) [17]. As a prerequisite for their clinical application, they need to be manufactured under standardized conditions and in compliance with EU guidelines for Good Manufacturing Practice (GMP) [18]. Yet, CD133^+^ stem cells for clinical use are purified using manual or semi-automatic devices based on the conventional magnetic cell sorting (MACS®) technique. Such purification requires high-level clean room facilities [19] and time-consuming logistics (Additional file 1: Figure S1a). Therefore, the development of an automatic system is urgently needed to guarantee stable product quality, to simplify the manufacturing procedure and to reduce the risk of contamination (Additional file 1: Figure S1b).

In this study, we evaluated the recently developed CE-marked CliniMACS Prodigy® BM-133 System (Prodigy) (Miltenyi Biotec GmbH, Bergisch Gladbach, Germany) in order to assess its suitability for the automatic manufacturing of a CD133^+^ cell product (CP) [20–22]. The entire manufacturing process, the examination of cell number and viability of the final CP as well as the depletion of non-target cells were part of the validation process, which was performed in compliance with EU guidelines for GMP. Furthermore, preclinical data were collected to compare proliferation and differentiation capacities of manually and automatically isolated CD133^+^ stem cells in accordance to Organisation for Economic Co-operation and Development (OECD) principles for Good Laboratory Practice (GLP) [23]. In addition, the efficacy of purified stem cells was assessed in immunodeficient mice 3 weeks after induction of myocardial infarction (MI).

## Methods

### Equipment and facility characteristics

The manufacturing process of the automatically generated CP was established at the Department of Cardiac Surgery (Rostock University Medical Center, Germany). The data were collected during the GMP inspection process, which led to the certification of GMP in compliance with EU guidelines (license DE_MV_01_MIA_2016_0001/310.0003.02), quality control (QC) and media fill validation were performed by the Institute for Clinical Chemistry and Laboratory Medicine (ILAB, Rostock University Medical Center) and the Institute for Medical Microbiology, Virology and Hygiene (IMIKRO, Rostock University Medical Center), which are accredited for compliance with clinical laboratory quality standards (ILAB: DIN EN ISO 15189, DIN EN ISO 22870, license D-ML-13193_02_00; IMIKRO: DIN EN ISO 15189, DIN EN ISO/IEC 17025). Data of in vitro colony-forming unit (CFU) assays were collected during the GLP evaluation process, which led to the statement of GLP compliance of the Reference and Translation Center for Cardiac Stem Cell Therapy (RTC). Therefore these experiments are closely aligned to the OECD principles for GLP [23] and the German Chemicals Act (ChemG §19a).

### BM aspiration

Sternal BM was aspirated from informed donors who gave their written consent to use their samples for research according to the Declaration of Helsinki. The ethics committee of Rostock University Medical Center has approved the presented study (registered as number A 2010 23) in 2010 (renewal in 2015). BM samples were obtained by sternal aspiration from patients undergoing coronary artery bypass graft (CABG) surgery at Rostock University Medical Center. Samples from six donors were used for automatic isolation and samples from another six donors for manual isolation. The mean volume of BM subjected to the automatic separation was 59.17 ± 2.6 ml (Additional file 2: Table S1a) with a mean donor age of 69 ± 4 years (including 83% of male donors) whereas for the manual isolation 56.5 ± 1.71 ml BM (Additional file 3: Table S2) with a mean donor age of 72 ± 2 years (including 100% of male donors) was used. Anticoagulation was achieved by heparinization with 250 IU/ml sodium heparin (Ratiopharm GmbH, Ulm, Germany).

### Manual cell isolation

Mononuclear cells from BM samples were isolated by density gradient centrifugation on Pancoll (PAN-Biotech GmbH, Aidenbach, Germany). CD133^+^ cells were enriched by positive magnetic selection using the MACS® cell separation system (Miltenyi Biotec). Labeling with direct anti-CD133 magnetic beads (Miltenyi Biotec) was performed as recommended by the manufacturer’s manual instructions. Total numbers of living cells were determined using trypan blue dye (0.4%, Sigma-Aldrich, Taufkirchen, Germany) and Neubauer hemocytometer (Carl Roth GmbH + Co. KG, Karlsruhe, Germany). Viability and frequency of CD133^+^ stem cells of BM and manually generated CPs were verified using BD LSRII flow cytometer and fluorescence-activated cell sorting (FACS) Diva software version 6.1.2 (Becton Dickinson, Heidelberg, Germany) (see below).

### Automatic cell isolation

All components of the Prodigy (consisting of the CliniMACS Prodigy® device, the CliniMACS Prodigy® Tubing Set (TS) 100, the CliniMACS® CD133 Reagent and the CliniMACS® Buffer (phosphate-buffered saline (PBS)/ethylenediaminetetraacetic acid (EDTA)) are CE-marked medical devices and were provided by Miltenyi Biotec. Medical products sodium chloride (NaCl, 0.9%) and human serum albumin (HSA, 20%) were obtained from Fresenius Kabi (Bad Homburg, Germany) and CSL Behring (Marburg, Germany), respectively.

By using the functionally closed Prodigy, CD133^+^ cells from sternal BM were purified in a mostly automatic procedure, which was performed by two operators under standardized conditions in compliance with EU guidelines for GMP (Additional file 4: Figure S2). For the isolation process, the BM-CD133 enrichment program was selected on the device. After installation of the TS 100, an integrity test was performed to control the leak-tightness of the TS. Subsequently, BM was applied to the system and after filtration an appropriate sample of non-diluted BM was taken into the BM pouch. To ensure automatic generation of plasma, BM aspirate was filled up with a fixed volume of 70 ml PBS/EDTA buffer supplemented with 20% HSA. Afterward, cell processing was performed following the selected enrichment program. Finally, the following fractions were obtained: CP (eluted in 0.9% NaCl supplemented with 10% autologous plasma), non-target cell bag (NTCB) and waste bag (WB). Next, all fraction bags were welded off and weighed with the consideration 1 g ≙ 1 ml. In an additional non-automatic step, 0.5 ml samples from the BM pouch and CP bag were taken for QC under aseptic conditions (laminar air flow, LAF). Samples of remaining fractions (NTCB, WB) were taken in some cases for further investigation (product quality review (PQR)).

### Prodigy media fill validation

In order to validate safety and reproducibility of the automatic aseptic manufacturing process in compliance with Ph. Eur. 5.1.1 as well as the relevant EU guidelines for GMP, a media fill validation [24] was performed. During this simulation BM, NaCl and CliniMACS® CD133 Reagent were replaced with Casein peptone Soybean flour peptone (CASO) Bouillon EP + USP medium (Merck Millipore, Darmstadt, Germany). At the end of the media fill process all manufactured samples (BM, CP) were transported to IMIKRO and tested for microbial growth according to Ph. Eur. 2.6.1/2.6.27 [24]. To verify the potential microbial growth, samples were incubated either at 35–37 °C under anaerobic or at 30–32 °C under aerobic conditions for 14 days. Simultaneously, the turbidity of the remaining NTCB and WB fractions was observed every day.

### Flow cytometric analysis of manually isolated cells

Frequency and viability of manually isolated CD133^+^ stem cells using a MACS® cell separation system were analyzed directly after the isolation procedure by flow cytometry. All utilized antibodies were mouse anti-human and are listed in Additional file 5: Table S3. Compensation was established using single-stained controls and gating was performed with matched isotype/fluorescence minus one (FMO) controls. The Boolean gating strategy for CD133^+^ cells was arranged on the basis of the International Society of Hematotherapy and Graft Engineering (ISHAGE) guidelines for CD34^+^ cell analysis [25] as described previously by our group [26].

Cell samples were suspended in MACS® buffer. FcR blocking reagent (Miltenyi Biotec) was added in order to reduce unspecific bindings. Subsequently, cells were incubated with antibodies for 10 min in the dark at 4 °C. To distinguish viable from dead cells, 7-amino-actinomycin (7-AAD) staining solution (BD) was used. After staining, red blood cell lysis buffer (eBioscience, Frankfurt am Main, Germany) was added and samples were incubated for 10 min on ice. Flow cytometry measurement was realized within the next 1 h by a BD™ LSRII flow cytometer. At least 1000 events were acquired within the final gate (viable CD45^+^/CD34^+^/CD133^+^ cells). This corresponds to the ISHAGE guidelines, which require a minimum of 100 events to be recorded in the target cell population. Analysis was performed using FACSDiva software (BD).

### QC and PQR of automatically generated fractions

After the automatic manufacturing process, BM, CP and PQR samples (NTCB, WB) were transported under controlled conditions (temperature: 24 °C; average time: 13 min) to ILAB and flow cytometric analysis were performed in compliance with EU guidelines for GMP. Cells were stained in duplicates with CD45-FITC/CD34-PE reagent and 7-AAD viability dye using IVD-certified Stem-Kit™ (Beckman Coulter (BC), Marseille, France) and with CD133-APC antibody (clone 293C2, APC conjugate, Miltenyi Biotec) (Additional file 5: Table S3). For each sample, a control staining with mouse isotype IgG2a-APC instead of CD133-APC was prepared. For calculation of cell numbers, 100 μl of a pre-mix of Stem Count Fluorospheres (included in the Stem-Kit™) was added to each tube. The measurement was performed using the Navios Flow Cytometer (BC) with scattergram analysis based on ISHAGE guidelines [25]. For QC, the CP was measured for 10 min in order to record at least 1000 events. A representative example of the gating strategy is shown in Additional file 6: Figure S5. The depletion of non-target cells [− log*CP*] according to Mohr et al. [27] was calculated as follows:

$$ - \log C P\kern0.5em  = - \log 10\frac{\left(\mathrm{viable}\ \mathrm{CD}{45}^{+}\mathrm{CP}-\mathrm{viable}\ \mathrm{CD}{133}^{+}\mathrm{CP}\right)}{\left(\mathrm{viable}\ \mathrm{CD}{45}^{+}\mathrm{BM}-\mathrm{viable}\ \mathrm{CD}{133}^{+}\mathrm{BM}\right)} $$

For PQR, thrombocyte and erythrocyte numbers were additionally analyzed using Sysmex XE-5000 (Sysmex Deutschland GmbH, Norderstedt, Germany).

The IMIKRO further tested microbial growth in CP, BM and PQR samples as described above (Prodigy media fill validation). Moreover, microbiological monitoring (LAF and fingerprints of the operator) was performed during the isolation process.

In order to evaluate the stability of the automatically generated CP over time, the CP bag was stored at room temperature (RT) for 2.5 h followed by 4 °C up to 24 h after manufacturing process and samples were taken for QC at the respective time points.

Samples used to investigate the biological activity of the CP were taken 2.5 h after the manufacturing process, since this represents a time span realistic for QC under optimal conditions.

### Acceptance criteria for the automatically generated CD133^+^ CP

Parameters selected to evaluate the quality of the automatically generated CD133^+^ CP using the Prodigy were based on the PERFECT clinical trial (clinicaltrials.gov, Identifier: NCT00950274). However, for ethical reasons in this study BM from the sternum served as a starting material while in the PERFECT clinical trial BM from the iliac crest was used. Therefore, acceptance criteria were correspondingly adapted. Briefly, a viability of ≥70%, a cellularity of 0.05 − 5 × 10^6^ viable CD133^+^ cells in 5 ml CP and depletion of non-target cells ≥2.5 log were considered as suitable.

### Expression of stemness-related proteins in the automatically generated CP

The expression of additional stemness (CD117, CD184, CD309) and maturation (CD14) markers in the CP was analyzed by flow cytometry in a multicolor panel as described previously by our group [26]. Data acquisition was performed based on ISHAGE gating strategy used for QC. All utilized antibodies were mouse anti-human and are listed in Additional file 5: Table S3.

1 × 10^4^ automatically enriched cells were suspended in MACS® buffer and FcR blocking reagent. Following this, cells were incubated with antibodies for 30 min in the dark at 4 °C. To distinguish viable from dead cells, LIVE/DEAD® Fixable Near-IR Dead Cell Stain Kit (Molecular Probes, Eugene, OR, USA) was used. Flow cytometry measurements were realized by BD™ LSRII flow cytometer and analysis was performed using FACSDiva software.

### Detection of apoptotic and necrotic cells in the automatically generated CP

In order to analyze the percentage of apoptotic and necrotic cells in the CP over a storage time of 24 h, the Annexin V Apoptosis Detection Kit (eBioscience) was performed in accordance to the manufacturer’s instructions. Annexin V – APC and 7-AAD were used to distinguish between early-stage apoptotic (Annexin V^+^), late-stage apoptotic (Annexin V^+^/7-AAD^+^), and necrotic (7-AAD^+^) cells.

### Hematopoietic colony-forming unit (CFU-H) assay

1 × 10^3^ CD133^+^ cells were plated in duplicates on a 35-mm dish in MethoCult H4434 Classic (Stemcell Technologies Inc., Vancouver, BC, Canada) 2.5 h after the manufacturing process following the manufacturer’s instructions in accordance with the OECD principles for GLP. After 14 days of incubation at 37 °C and 5% CO_2_, formed colonies were scored as CFU-erythroid (CFU-E), burst-forming unit-erythroid (BFU-E), CFU-granulocyte, macrophage (CFU-GM) and CFU-granulocyte, erythrocyte, macrophage, megakaryocyte (CFU-GEMM).

### Colony-forming unit endothelial cell (CFU-EC) assay

1 × 10^3^ CD133^+^ cells were plated in triplicates on a 35-mm dish in supplemented MethoCult SF H4236 (Stemcell Technologies Inc.) 2.5 h after the manufacturing process as previously described [28] in accordance to OECD principles for GLP. After 14 days of incubation at 37 °C and 5% CO_2_, adherent and non-adherent colonies were counted.

### Immunofluorescence staining of non-adherent cells from CFU-EC assay

In order to confirm the endothelial phenotype of non-adherent cells immunofluorescence staining was performed. Therefore, non-adherent cells obtained from the CFU-EC assay were collected after 29 days of incubation by dissolving the supplemented MethoCult SF H4236 with 1 U/ml dispase (Stemcell Technologies Inc.). Cells were counted by TC20™ Automated Cell Counter (Bio-Rad Laboratories Inc., Hercules, CA, USA) and 1.5 × 10^5^ were cultured for 3 additional days in Endothelial Cell Growth Medium (EGM)-2 (Lonza Group Ltd., Basel, Switzerland) on fibronectin-coated coverslips (Human Plasma Fibronectin from Merck Millipore, 10 μg/ml in PBS).

For uptake analyses of acetylated low-density lipoprotein (acLDL), cells on coverslips were incubated with 10 μg/ml of Alexa Fluor 488-labeled acLDL antibody (Thermo Fisher Scientific, Schwerte, Germany) for 1 h at 37 °C. Subsequently, cells were fixed with 4% paraformaldehyde (PFA, Merck Millipore) and nuclei were stained with Hoechst dye (1:1000, Sigma-Aldrich).

For immunostaining of von Willebrand factor (vWF) cells were fixed with 4% PFA, permeabilized with 0.1% Triton X-100 (Sigma-Aldrich) for 7 min at RT and blocked with 1% bovine serum albumin (BSA, Sigma-Aldrich) in PBS for 1 h at RT. Afterward, cells were stained with rabbit polyclonal vWF primary antibody (1:50, Santa Cruz Biotechnology Inc., Dallas, TX, USA) overnight at 4 °C, goat anti-rabbit Alexa Fluor 488 secondary antibody (1:350, Thermo Fisher Scientific) for 3 h at 37 °C and counterstained with Hoechst dye (1:1000, Sigma-Aldrich).

Finally, all coverslips were mounted on a microscope slide using FluorSave™ (Merck Millipore) and pictures were taken and analyzed using ELYRA PS.1 LSM 780 microscope and ZEN 2011 software (Carl Zeiss GmbH, Jena, Germany). For acLDL uptake and vWF expression, tiles scans of 850 μm x 850 μm were acquired using the × 40 objective.

### Experimental design of the animal model

The federal animal care committee of Landesamt für Landwirtschaft, Lebensmittelsicherheit und Fischerei Mecklenburg-Vorpommern (LALLF M-V, Germany) approved the study protocol (registered as number LALLF M-V/TSD/7221.3-1.1-088/11). Severe combined immunodeficient beige mice (SCID bg; strain CB17.Cg-*Prkdc* ^*scid*^ *Lyst* ^*bg-J*^/Crl) were purchased from Charles River Laboratories (Sulzfeld, Germany). SCID bg mice (female, 22 ± 2 g) were randomly assigned to four groups: healthy control group (SHAM), two MI groups with human CD133^+^ stem cell treatment of the respective method (MI133 manual, MI133 automatic) and untreated MI control group (MIC).

### Generation of reperfused MI and intramyocardial stem cell implantation

Mice were anesthetized with pentobarbital (50 mg/kg, intraperitoneal). After thoracotomy and preparation, the left anterior descending coronary artery (LAD) was ligated. After 45 min each mouse received an intramyocardial injection. For cell treatment 1 × 10^5^ CD133^+^ stem cells were suspended in 10 μl of MACS® buffer (PBS (PAN-Biotech GmbH) supplemented with 0.5% BSA and 2 mM EDTA (Thermo Fisher Scientific)) and mixed with an equal amount of BD Matrigel™ Matrix 2.5 h after the manufacturing process. Both control groups underwent the same approach without cells. Injections of 4 × 5 μl were given along the border of the blanched myocardium and ligation was removed. SHAM-operated mice underwent identical surgical procedures without LAD ligation.

### Left ventricular catheterization

Three weeks after surgery, mice underwent pressure-volume (PV) loop measurements according to the protocol of CardioDynamics BV (CD Leycom, Zoetermeer, Netherlands). Data were collected with the Millar Pressure-Volume System (consisting of the Ultra-Miniature Pressure-Volume Catheter (model SPR-1030), the Pressure Conductance Unit (model MPCU-200) and the PowerLab data-acquisition hardware). Calibration of pressure and volume was performed by equating the minimal and maximal conductance with minimal (0 mmHg) and maximal (100 mmHg) pressures as well as minimal and maximal blood volumes received from venous circulation. After inserting the catheter into the carotid artery, retrograde access to the left ventricle (LV) was achieved. PV loops were recorded under normal conditions (baseline) followed by stress conditions mediated by intravenous dobutamine administration (10 μg/kg/min, Sigma-Aldrich). Volume signal was corrected by measurement of wall conductance (parallel volume) via hypertonic saline (5%) injection. Data were analyzed with IOX Version 1.8.3.20 software (Emka Technologies, Paris, France). After PV loop measurements blood vessels were stained with 7.5 μg/ml of biotinylated *Lycopersicon esculentum* (tomato) lectin (LINARIS, Wertheim-Bettingen, Germany) by perfusion of the venous circulation for 10 min. For euthanization hearts were arrested in diastole with potassium chloride.

### Organ harvesting

Each heart was removed, embedded in O.C.T.™ Compound (Tissue-Tek®; Sakura Finetek, Zoeterwoude, Netherlands) and snap-frozen in liquid nitrogen. For histological and biomolecular investigations the infarct area of heart tissue has been divided into four horizontal levels from the apex to the base and within each sections of 5 μm were cut.

### Infarction size and fibrosis

Heart sections of four horizontal infarction levels (5 μm) were stained with Sirius Red (Division Chroma, Muenster, Germany) visualizing collagen deposition and Fast Green FCF (Sigma-Aldrich) displaying uninjured muscle tissue. To investigate the infarction size, two contiguous levels of the heart, which represent the major infarction ratio, were analyzed using computerized planimetry (Axio Vision LE Rel. 4.5 software; Carl Zeiss GmbH). To evaluate fibrosis, the Sirius Red-positive regions of collagen deposition in the infarction border zone (BZ) and remote area (RA) were examined in five randomly chosen fields (each per section; one section per level) using computerized planimetry. Collagen deposition was expressed as the ratio of collagen deposition to myocardial tissue in percentage.

### Determination of blood vessels

Tomato lectin perfusion of the hearts as described was used for analysis of capillary density and angiogenesis. Heart sections of two contiguous levels of the heart, which represent the major infarction region, were fixed with 4% PFA and immunostained with polyclonal goat anti-biotin (Vector Laboratories; Burlingame, CA, USA) primary antibody followed by anti-goat Alexa-Fluor 488 (Molecular Probes™/Thermo Fisher Scientific) conjugated secondary antibody and counterstained with 4′,6-diamidino-2-phenylindole (DAPI; Sigma-Aldrich). The sections were analyzed within the BZ, RA and infarcted scar (IS) of the heart. Capillary density as well as neovascularization were assessed by counting the number of capillaries in five BZ, RA and IS randomly chosen fields per section (one section per level). Results were expressed as capillaries per high power field (HPF).

### Statistical analysis

Statistical analysis was performed by Student’s *t* test with SigmaPlot version 11.0 (Systat Software Inc., Chicago, IL, USA). For analysis of possible correlation of normally distributed variables, Pearson product-moment was used. All values are presented as mean ± standard error of the mean (SEM). *P* values ≤ 0.05 (*); ≤ 0.01 (**); and ≤ 0.001 (***) were considered as statistically significant.

## Results

### The Prodigy is a convenient tool to simplify and standardize the manufacturing procedure of CPs

In this study, the whole manufacturing procedure of the CD133^+^ CP (isolation, transport and QC) was established on-site and in compliance with EU guidelines for GMP using the Prodigy. Therefore, our hospital (Rostock University Medical Center, Germany) has received for the first time the certificate of GMP compliance of a manufacturer (license DE_MV_01_MIA_2016_0001/310.0003.02) for this device.

Usage of the Prodigy enables purification of the CP within approximately 4 h and requires only few interactions of the operators (Additional file 4: Figure S2). This will further diminish the risk of contamination and will minimize inter-individual variability caused by the manufacturing personnel, thereby resulting in higher standardized product quality. Moreover, the entire on-site manufacturing enables reduction of logistical efforts, which in turn leads to a shorter hospitalization time of patients and thereby also diminished costs.

### The Prodigy is suitable for the automatic generation of a CD133^+^ CP

**Fig. 1:**
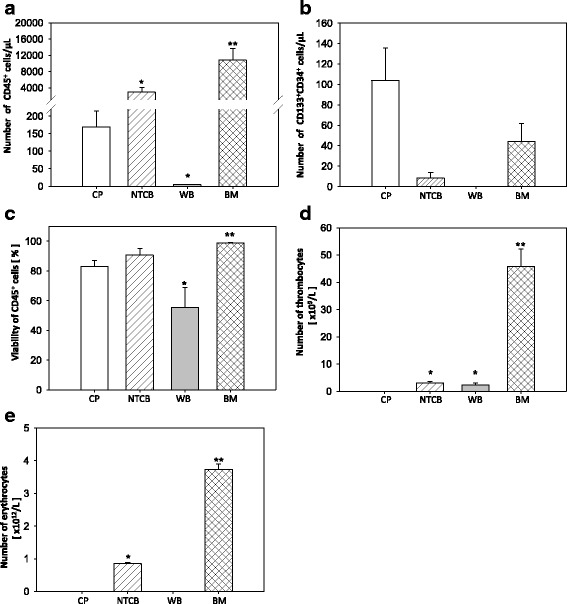
Characterization of automatically generated fractions. Cell product (CP), non-target cell bag (NTCB), waste bag (WB) and bone marrow (BM) were analyzed in respect of their CD45^+^ cells/μl (**a**), CD133^+^CD34^+^ cells/μl (**b**) and viability of CD45^+^ cells (**c**) using flow cytometry measurement in accordance with ISHAGE guidelines. Additionally, total numbers of thrombocytes (**d**) and erythrocytes (**e**) were assessed using a Sysmex device. All data are presented as mean ± SEM. CP, BM (*n* = 6); NTCB, WB (*n* = 3). *p ≤ 0.05; **p ≤ 0.01; ***p ≤ 0.001 vs. CP

Initially, we characterized the automatically generated CD133^+^ CPs by evaluating QC-relevant parameters. The CPs contained on average 0.52 × 10^6^ ± 0.16 CD133^+^ stem cells per 5 ml with a viability of 83.13 ± 3.9% and a mean log_10_ depletion of 3.23 ± 0.19 was obtained, which is in accordance to defined acceptance criteria (Table 1 and Fig. 1c). Based on ISHAGE guidelines we calculated that 64.0 ± 8.8% of cells in the CP were CD45^+^/CD34^+^/CD133^+^ target cells (Additional file 2: Table S1a). Importantly, the presence of thrombocytes, erythrocytes and lymphocytes in the CP can be excluded based on results from full blood counts (Sysmex analyses) and flow cytometric measurements (Fig. 1d and e; Additional file 6: Figure S5h). Reanalysis of the quality control data demonstrated that the non-target cell fraction (36.4% of the CP) consists of CD45^+^/CD34^-/low^/CD133^−^ (28.8%) and CD45^+^/CD34^+^/CD133^−^ (7.6%) cells (regions J, Q and I in Additional file 6: Figure S5b and e). Importantly, the entire non-target population is characterized by a hematopoietic phenotype due to its expression of the CD45 marker. Moreover, CD45^+^/CD34^low^/CD133^−^ (region Q) and CD45^+^/CD34^+^/CD133^−^ (region I) cells were found to have similar size and granularity as the target cells (>90%; regions S and P in the Additional file 7: Figure S6). The analysis of various additional stem/progenitor cell markers demonstrated that the non-target cell fraction (CD45^+^/CD34^−^/CD133^−^) (region J) is characterized by the lowest expression of CD117 as well as the highest expression of CD184 (CXCR4) and CD309 (KDR, VEGFR2) in comparison to all other non-target and target cell fractions (Additional file 8: Table S4). Additionally, this fraction showed also the highest expression of the maturation marker CD14.

**Table 1:** Acceptance criteria of the automatically generated cell product (CP)

Validation no.	No. of viable CD133^+^cells (×10^6^) in 5 ml	Cell viability [%]	Depletion of non-target cells [−log CP]
1	1.0	97.92	3.86
2	0.28	77.17	2.82
3	0.57	71.42	2.60
4	0.19	87.13	3.46
5	0.12	77.66	3.49
6	0.97	87.53	3.17
Mean	0.52	83.14	3.23
SEM	0.16	3.90	0.19
Acceptance range	0.05–5 × 10^6^	≥70%	≥2.5 log

Number of viable CD133^+^ cells and cell viability of the automatically generated CP were analyzed by flow cytometric analysis in accordance with ISHAGE guidelines. Depletion of non-target cells was calculated as the negative logarithm to base 10 of number of total CD133^+^ cells in CP divided by the number of CD133^+^ cells in bone marrow. All data are presented as mean ± SEM

**Fig. 2:**
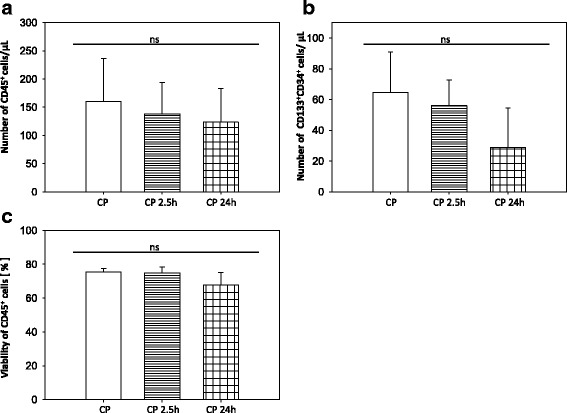
Stability of the automatically generated cell product (CP) over a storage time of 2.5 h and 24 h post manufacturing process. For the first 2.5 h, CP was stored under room temperature (RT) conditions. After 2.5 h CP was stored at 2–8 °C. Samples of CP were taken at the respective storage time and CD45^+^ cells/μl (**a**), CD133^+^CD34^+^ cells/μl (**b**) and viability of CD45^+^ cells (**c**) were measured by flow cytometry in accordance with ISHAGE guidelines. All data are presented as a mean ± SEM. *n* = 3. *p ≤ 0.05; **p ≤ 0.01; ***p ≤ 0.001 vs. CP

The stability analysis of the CP showed no significant changes over the entire storage time in CD45^+^ and CD133^+^/CD34^+^ cells per μl and cell viability (Fig. 2). However, a trend to decrease was detectable for cells per μl after 24 h. Moreover, no significant changes in the percentage of apoptotic and necrotic cells for up to 24 h post manufacturing were detected (Additional file 9: Figure S7). In addition, all manufactured CPs were free from microbial contamination as proven by microbiological controls.

For characterization of all automatically generated fractions mean volumes were determined by visual control or by weighing. All volumes showed small SEMs confirming the successful standardization of the manufacturing process (Additional file 2: Table S1a and b). In comparison to CP the number of CD45^+^ cells per μl was significantly higher in BM as well as NTCB, while it was significantly lower in WB (Fig. 1a). Importantly, no target cells were detected in WB (Fig. 1b; Additional file 2: Table S1b). This indicates that most of the hematopoietic cells were transferred from BM (starting material) to the NTCB. The highest CD133^+^CD34^+^ cell number per μl in CP demonstrates the efficient enrichment of CD133^+^ stem cells by the Prodigy. The cell viability of CP and NTCB (83.14 ± 3.90% and 90.65 ± 4.36%) were comparable. BM showed slightly but significantly higher cell viability (98.66 ± 0.36%) in comparison to CP and NTCB, indicating the mild manufacturing conditions. Additionally, the viability of CP did not correlate with the viability of the starting material (Pearson correlation: *r* = 0.288; *p* = 0.580). The microbiological monitoring of all validation runs resulted in <1 CFU, showing that all processes were conducted under standardized aseptic conditions. Overall, the manufacturing process lasted 3 h 27 min ± 12 min.

### Manually and automatically isolated CD133^+^ cells bear similar hematopoietic and endothelial differentiation capacity

**Fig. 3:**
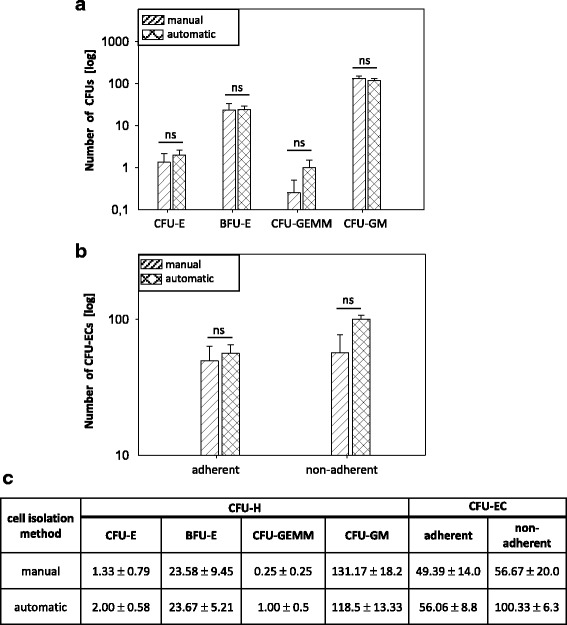
Hematopoietic and endothelial differentiation capacity of manually and automatically isolated CD133^+^ stem cells. Hematopoietic colony-forming unit (CFU-H) and colony-forming unit endothelial cells (CFU-EC) assays were performed by seeding 1 × 10^3^ cells directly after the isolation procedure. Hematopoietic CFUs (CFU-E, BFU-E, CFU-GEMM, CFU-GM) (**a**) and endothelial CFUs (adherent, non-adherent) (**b**) were counted after 14 days of incubation. All numbers of counted CFUs are presented in the table (**c**). All data are presented as a mean ± SEM. *n* = 3. *p ≤ 0.05; **p ≤ 0.01; ***p ≤ 0.001

BM-derived CD133^+^ stem cells are known to bear multipotent differentiation potential. In order to detect the influence of the isolation process on their hematopoietic and endothelial differentiation capacity, CFU-H and CFU-EC assays were performed. The comparison of manually and automatically isolated cells showed neither a significant difference in the amount of formed hematopoietic CFUs (CFU-E, BFU-E, CFU-GEMM, CFU-GM) nor in the amount of formed endothelial CFUs (adherent, non-adherent) (Fig. 3). Immunofluorescence staining demonstrated similar acLDL uptake (Additional file 10: Figure S3) and vWF expression (Additional file 11: Figure S4) of manually and automatically enriched CPs, confirming their equal endothelial differentiation potential.

### Manually and automatically isolated CD133^+^ cells bear similar cardiac regeneration potential

**Fig. 4:**
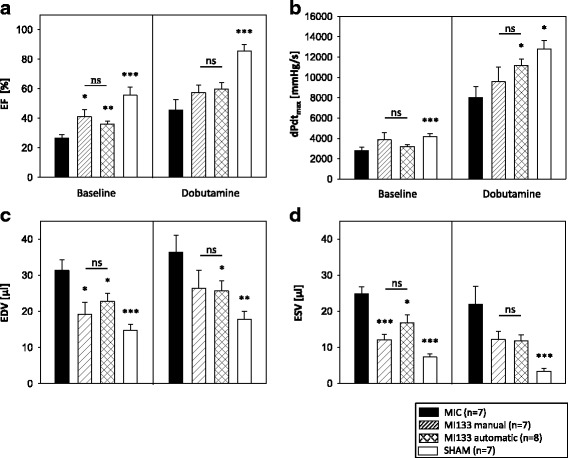
Comparison of the cardiac regeneration potential of manually and automatically isolated CD133^+^ stem cells. 1 × 10^5^ CD133^+^ stem cells were intramyocardially transplanted into SCID bg mice after myocardial infarction (MI). Ejection fraction (EF) (**a**), velocity of pressure rise (dPdt_max_) (**b**), end-diastolic volume (EDV) (**c**), and end-systolic volume (ESV) (**d**) were assessed by pressure-volume (PV) loop measurements 3 weeks after cell transplantation. For control untreated infarction (MIC) and SHAM operation were used. All parameters were measured under baseline and under stress conditions mediated by intravenous dobutamine administration (10 μg/kg/min). Data are presented as mean ± SEM. *p ≤ 0.05; **p ≤ 0.01; ***p ≤ 0.001 vs. MIC; *ns* not significant

In order to assess the cardiac regenerative potential of manually and automatically generated CD133^+^ CPs, cells were transplanted into SCID bg mice after cardiac ischemia/reperfusion. Cardiac performance was analyzed by hemodynamic measurement 3 weeks after cell transplantation. PV loops demonstrate significant improvement of ejection fraction (EF), end-diastolic volume (EDV) and end-systolic volume (ESV) under baseline condition in comparison to the untreated infarction (MIC) after manually and automatically generated CD133^+^ stem cell transplantation (Fig. 4). Under stress conditions velocity of pressure rise (dPdt_max_) and ESV were significantly improved in comparison to MIC after transplantation of automatically enriched CD133^+^ cells. Importantly, no significant differences in cardiac regeneration potential were obtained between manually and automatically isolated CD133^+^ stem cells.

### Manually and automatically isolated CD133^+^ cells positively influenced cardiac remodeling

**Fig. 5:**
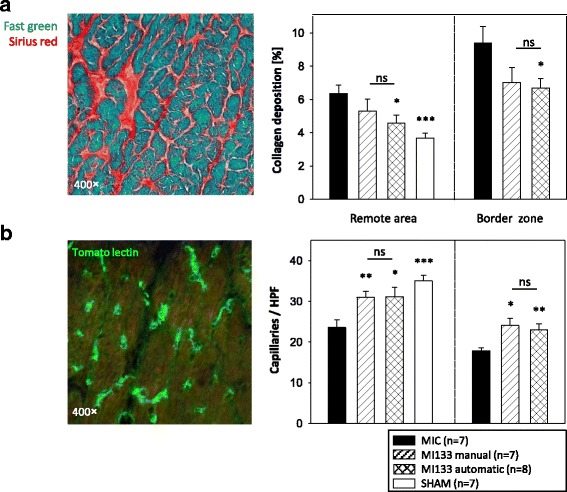
Effects of manually and automatically isolated CD133^+^ stem cells on cardiac remodeling. 1 × 10^5^ CD133^+^ stem cells were intramyocardially transplanted into SCID bg mice after myocardial infarction (MI). For evaluation of histological changes, fibrotic events (**a**) and decrease of capillary density (**b**) at infarction border zone and remote area were analyzed 3 weeks after transplantation. Animals with untreated infarction (MIC) and SHAM operation were used as control groups. Data are presented as mean ± SEM. *p ≤ 0.05; **p ≤ 0.01; ***p ≤ 0.001 vs. MIC; *ns* not significant

Ligation of the LAD consistently resulted in a transmural MI with its typical histologic changes including the thinning of the left ventricular free wall, extensive collagen deposition (fibrosis) and decrease of capillary density following hypertrophy of the cardiac cells 3 weeks after infarction. The transplantation of automatically isolated CD133^+^ stem cells led to a significant decrease in fibrosis (versus MIC) in both RA and BZ (Fig. 5a). Manually isolated stem cells also resulted in a slightly but not significantly reduced collagen deposition in comparison to MIC. Moreover, explanted hearts were perfused with biotinylated tomato lectin followed by anti-biotin staining of the heart cryosections. Decrease of capillary density was significantly reduced after stem cell treatment in both RA and BZ in comparison to MIC (Fig. 5b).

### Manually and automatically isolated CD133^+^ cells have beneficial effects on formation of the infarction scar

**Fig. 6:**
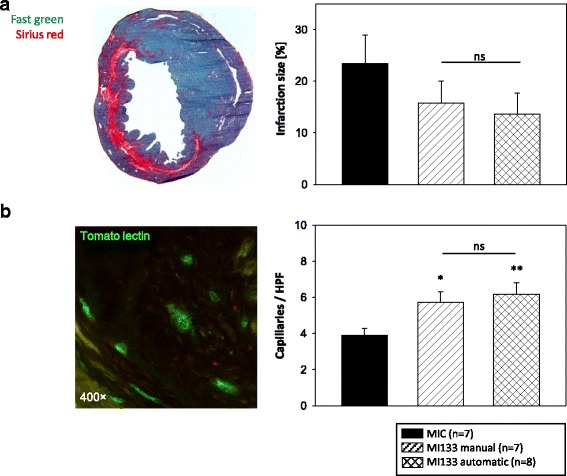
Effects of manually and automatically isolated CD133^+^ stem cells on formation of infarction scar. 1 × 10^5^ CD133^+^ stem cells were intramyocardially transplanted into SCID bg mice after myocardial infarction (MI). Three weeks after cell transplantation infarction size (**a**) and infiltration of the infarcted scar with new blood vessels (**b**) were analyzed. For control untreated infarction (MIC) and SHAM operation were used. Data are presented as mean ± SEM. *p ≤ 0.05; **p ≤ 0.01; ***p ≤ 0.001 vs. MIC; *ns* not significant

Planimetry measurements showed a trend toward reduction in infarction size after transplantation of stem cells derived by either isolation procedure compared to MIC (Fig. 6a). Neovascularization in the infarct scar was significantly improved after stem cell application (versus MIC) (Fig. 6b). No significant differences were observed between manually and automatically isolated CD133^+^ stem cells.

## Discussion

Therapeutic benefits of transplanted CD133^+^ stem cells in chronic ischemic cardiomyopathy have been established for years [5–9]. In 2011, our autologous BM-derived CD133^+^ CP intended for the regeneration of ischemic heart tissue was defined as a tissue-engineered product and classified as ATMP by the EMA. For clinical translation, the manufacturing of ATMPs requires specific standards of quality [18, 29]. The recently developed CE-marked Prodigy allows the automatic on-site purification of CD133^+^ stem cells in a GMP-qualified ambit under reduced clean room requirements. This closed system reduces the risk of contamination, minimizes inter-individual variability caused by the manufacturing personnel and therefore enables a higher standardized product quality compared to conventional semi-automatic isolation systems.

In this study we demonstrate that the Prodigy is a suitable tool for automatic manufacturing of a CD133^+^ CP from human BM in compliance with EU guidelines for GMP. The manufactured CPs were free of microbial contaminations and fulfilled all defined acceptance criteria including cellularity, viability and depletion of non-target cells. Furthermore, we could show that automatically isolated cells bear proliferation, differentiation and regeneration capacities comparable to manually isolated cells.

On average 0.52 × 10^6^ viable CD133^+^ cells were isolated from 59 ml sternal BM automatically in 5 ml CP using the Prodigy within approximately 4 h. Noticeably, this number of CD133^+^ cells varied in a range from 0.12 × 10^6^ to 1 × 10^6^. One reason for this relatively wide variety might be individual cardiovascular risk factors of BM donors undergoing CABG surgery [30], which have been shown to affect the percentage of BM-derived progenitor cells [31–33]. Moreover, variations in BM quality caused by dilution with peripheral blood can influence the number of CD133^+^ stem cells in the CP. However, CD133^+^ cell amounts varied in the range of our acceptance criteria considering those inter-individual fluctuations. Furthermore, results demonstrated a viability of >80% and a depletion of 99.9% of non-target cells. While this depletion is calculated on hematopoietic nucleated cells alone, it is important to note that thrombocytes as well as erythrocytes were completely absent from the CP. In 2014 the EMA defined presence of granulocytes and erythrocytes as a significant impurity in CPs because they influence the functionality of target progenitor cells and affect the LVEF recovery in a negative way [34]. Therefore, it is a prerequisite to keep the amount of these cells to a minimum. In addition, flow cytometric measurements revealed that the entire non-target cell population is characterized by a hematopoietic phenotype as evident from its expression of CD45. We assume that within this non-target population one fraction (CD45^+^/CD34^low/+^/CD133^−^) represents hematopoietic stem and progenitor cells [35]. The second non-target fraction (CD45^+^/CD34^−^/CD133^−^) seems to be in a more progressed stage of maturation as indicated by a lower expression of the stem cell marker CD117 and a higher expression of the maturation marker CD14 [26]. Moreover, this fraction shows the highest expression of CD184 (CXCR4) as well as CD309 (KDR, VEGFR2) indicating a possible role in stem cell homing and in the regulation of angiogenesis, respectively [30, 36, 37]. Therefore, we suggest that the non-target cells likely represent a mixture of phenotypically and functionally distinct stem cells and progenitors. Another important observation is the loss of approximately half of the target CD133^+^ stem cells, which were transferred into NTCB. This limitation was also observed by Stroncek et al. [38], who suggested that large quantities of platelets can interfere with the antibody-linked paramagnetic particle binding to hematopoietic antigens. Additionally, this might be explained by the low expression of the CD133 antigen on the surface of these cells [34]. Moreover, our results demonstrate that the automatic manufacturing procedure results in a significantly lower viability and frequency of CD133^+^ cells in the CP when compared to manual manufacturing. This can be explained by the fact that the manual isolation protocol includes additional centrifugation steps eliminating dead cells and debris, as well as substances increasing the purity of the CP (e.g., Pancoll, FcR blocking reagent) – however, none of these reagents is suitable for clinical application. Therefore, the Prodigy is currently the only available device enabling automatic manufacturing of a high-quality CP which fulfills all acceptance criteria and is suitable for clinical translation.

The time lag before application of ATMPs caused by the QC and release processes after their manufacturing may pose a serious limitation for clinical use. In this study, the CP was stored at RT directly after the isolation process for 2.5 h to ensure stable cell temperature conditions during the whole manufacturing procedure (isolation, transport, QC, and storage time). However, since cells typically show metabolic activity at RT, these storage conditions (0.9% NaCl supplemented with autologous plasma) are not suitable for longer time periods. Therefore, the CP was stored at 4 °C after the initial 2.5 h in order to minimize the cell metabolism [39].

After a storage time of 24 h, there was a clear tendency toward a decrease of the CD133^+^CD34^+^ cell numbers per microliter. Yet, this phenomenon of reduced CD133^+^CD34^+^ cells is due to reduced CD34 and CD133 protein expression over time as previously described by our group [28]. Importantly, we did not find any significant changes in the percentage of apoptotic and necrotic cells for up to 24 h post manufacturing. This demonstrates the suitability of the automatically generated CD133^+^ CP for clinical application even 24 h post manufacturing. Our results are in agreement with previous data of Belotti et al., where a stable quality of CD133^+^ cells was reported for up to 12 h at 2–8 °C in serum-free basal medium [31]. In 2014 our group recommended a maximal storage time of 30 h at 2–8 °C for hematopoietic stem cells [28]. However, keeping the storage time as short as possible is of course always preferable to avoid cell loss.

In order to ensure the biological activity of the automatically generated CD133^+^ CPs 2.5 h post manufacturing, we applied different in vitro and in vivo assays. For evaluation, we compared the obtained data with manually isolated CD133^+^ cells, which have been used in several preclinical [13, 30] and clinical [7, 40, 41] studies for cardiac regeneration and served as an experimental model, here. In vitro differentiation assays were performed in order to investigate the regeneration potential of CD133^+^ stem cells, which is based on their secretion of paracrine factors and their direct participation into neovascularization [14, 16]. Thereby, we did neither detect significant differences in endothelial nor in hematopoietic differentiation capacities between automatically and manually isolated CD133^+^ cells. This indicates that the multipotent potential of these cells is not affected by the Prodigy manufacturing process. To confirm therapeutic effects of CD133^+^ cells in vivo, heart function was assessed in mice after induction of MI. After transplantation of manually isolated CD133^+^ cells a significant improvement in cardiac function was detected compared to MIC, which is in accordance with previous findings of our [13] and other groups [42]. Importantly, for the first time we also could demonstrate that automatically isolated CD133^+^ cells led to a significant increase in different cardiac function parameters in vivo. Additionally, our data revealed that the automatically isolated CD133^+^ CPs were actively involved in the reduction of the cardiac remodeling process and participated in neovascularization at a level comparable to manually isolated cells.

## Conclusions

Our data demonstrate that the automatic Prodigy is a suitable system for safe and time-saving on-site manufacturing of a high-quality BM-derived CD133^+^ CP in a GMP-qualified ambit intended for the treatment of ischemic heart disease. The manufacturing process was performed in compliance with EU guidelines for GMP and the purified CD133^+^ CP fulfilled all defined acceptance criteria, which were adapted from our currently running phase III clinical trial PERFECT. The obtained data were part of the GMP evaluation process by the competent local authority, which granted the manufacturing license for our cell manufacturing facility. Moreover, in vitro and in vivo generated preclinical data evidenced safety, biological activity, and cardiac regeneration potential of purified cells, which is a prerequisite for the initiation of first clinical trials applying an automatically generated CD133^+^ CP using the Prodigy.

## Additional files

Additional file 1: Figure S1. Schematic representation of two different logistical procedures used for the manufacturing of stem cell products in cardiovascular regenerative medicine. Manual/semi-automatic systems require centralized GMP-compliant manufacturing by an external service provider (a). Entirely closed systems such as the automatic CliniMACS Prodigy® enable decentralized GMP-compliant manufacturing by an in-house facility (b). a Centralized GMP-compliant manufacturing by an external service provider: few specialized contractors are available for a distinct cell product because standardized high-level clean rooms (Class A in B) and sophisticated training are required for the manual or semi-automatic manufacturing. This results in higher costs of the therapy due to an increased logistical effort (e.g., long-distance transportation) and longer hospitalization of the patients as well as a possible loss of cell product quality (e.g., CliniMACS® Plus). b Decentralized GMP-compliant manufacturing by an in-house facility: cell products intended for clinical application can be manufactured on-site owing to reduced clean room requirements (Class A in D) and easier standardization of processes with minimized inter-individual variability due to automatic manufacturing. Lower logistical effort (in-house transport) and shorter hospitalization enables minimized costs plus a better and stable quality of the cell product (e.g., CliniMACS Prodigy®). (PDF 109 kb)
Additional file 2: Table S1. Overview of data obtained from CliniMACS® Prodigy BM-133 System validation process (a) and product quality review (PQR) (b). Volumes of samples were ascertained by visual control (bone marrow (BM)) or by weighing (cell product (CP), non-target cell bag (NTCB), waste bag (WB)). Total numbers of viable CD45^+^ and CD133^+^ cells were calculated by multiplying absolute cell numbers per volume unit with corresponding volumes. The frequencies of viable CD133^+^ cells were measured by flow cytometry in accordance with ISHAGE guidelines. (DOC 42 kb)
Additional file 3: Table S2. Overview of data obtained from manual cell isolation. Volumes of bone marrow (BM) samples were ascertained by visual control. Total numbers of viable CD133^+^ cells were determined using Neubauer hemocytometer. The frequencies of viable CD133^+^ cells were measured by flow cytometry in accordance with ISHAGE guidelines. (DOC 36 kb)
Additional file 4: Figure S2. Schematic schedule of the CD133^+^ cell isolation process using the CliniMACS Prodigy® BM-133 system. *Gray*: manual steps (two operators); *green*: semi-automatic steps (one operator, dual control); *blue*: automatic steps. (PDF 1368 kb)
Additional file 5: Table S3. List of antibodies used for flow cytometry-based quality control of manually isolated CD133^+^ stem cells and for the characterization of stemness marker expression in the automatically generated cell product. (DOC 33 kb)
Additional file 6: Figure S5. Representative ISHAGE-based gating strategy used for the quality control of the automatically generated cell product (CP). Debris was excluded from CD45^+^ cells (a). CD34^+^ cells were selected from viable CD45^+^ cells (b). Events with high expression of the CD45 marker were excluded from viable CD45^+^/CD34^+^cells (c). FSC/SSC backgate was employed to select viable CD45^+^/CD34^+^ hematopoietic progenitor cells (HPCs) with blast morphology (d). Viable CD45^+^/CD34^+^/CD133^+^ cells were selected (e). Events with high expression of the CD45 marker were excluded from viable CD45^+^/CD34^+^/CD133^+^ cells (f). FSC/SSC backgate was employed to select viable CD45^+^/CD34^+^/CD133^+^ HPCs with blast morphology (g). A control gate (‘Ly’, lymphocytes) was used during exclusion of mature CD45^+^ HSCs. *Red*: target cell population. *Gray*: dead cells. (PDF 374 kb)
Additional file 7: Figure S6. Phenotypic and morphologic characterization of non-target cell populations presented in the automatically generated cell product (CP). Non-target cell populations were subdivided into three fractions: CD45^+^/CD34^−^ (*region J*) and CD45^+^/CD34^low^ (*region Q*) (a); CD45^+^/CD34^+^/CD133^−^ (*region I*) (b). CD45^+^/CD34^-/low^ non-target cells were further analyzed concerning their CD133 expression (*regions M and R*, respectively). Size and granularity of all three fractions were evaluated: *region K and T*: smaller than target cell population; *region O, S and P*: same size/granularity than target cell population; *region L*: higher granularity than target cell population. (PDF 130 kb)
Additional file 8: Table S4. Stemness marker expression of target and non-target cell fractions presented in the automatically generated cell product (CP). For phenotype characterization the expression of stemness markers (CD117, CD184, CD309, CD14) was evaluated in target cells (CD45^+^CD34^+^CD133^+^) (a) and non-target cells (CD45^+^CD34^−^CD133^−^, CD45^+^CD34^low^CD133^−^, CD45^+^CD34^+^CD133^−^) (b) using flow cytometry. The respective analysis was made in accordance with ISHAGE guidelines. Data are presented as a mean ± SEM, *n* = 9. (DOC 37 kb)
Additional file 9: Figure S7. Determination of apoptotic and necrotic cells in the automatically generated cell product (CP) after storage. The percentage of viable, early-stage apoptotic, late-stage apoptotic and necrotic cells was analyzed by flow cytometric measurements using Annexin V Apoptosis Detection Kit. Samples of CP were taken at the respective storage time. All data are presented as a mean ± SEM. *n* = 3. **p* ≤ 0.05; ***p* ≤ 0.01; ****p* ≤ 0.001. (PDF 108 kb)
Additional file 10: Figure S3. Uptake of acetylated low-density lipoprotein (acLDL) of colony-forming unit endothelial cell (CFU-EC) assay-derived cells. CFU-EC assay was performed and after 29 days incubation the uptake of acLDL (*green*) was examined by immunostaining in non-adherent cells. Nuclei were stained with Hoechst dye (*blue*). Pictures were taken using ELYRA PS.1 LSM 780 microscope (Carl Zeiss GmbH). Images of manually (a and b) and automatically (c and d) isolated cells. Scale bars: 100 μm (a and c) and 20 μm (b and d). (PDF 341 kb)
Additional file 11: Figure S4. Von Willebrand factor (vWF) expression of colony-forming unit endothelial cell (CFU-EC) assay-derived cells. CFU-EC assay was performed and after 29 days incubation the vWF expression (*green*) was examined by immunostaining in non-adherent cells. Nuclei were stained with Hoechst dye (*blue*). Pictures were taken using ELYRA PS.1 LSM 780 microscope (Carl Zeiss GmbH). Images of manually (a and b) and automatically (c and d) isolated cells. Scale bars: 100 μm (a and c) and 20 μm (b and d). (PDF 295 kb)

## Abbreviations

7-AAD: 7-amino-actinomycin
acLDL: acetylated low-density lipoprotein
ATMP: advanced therapy medicinal product
BFU-E: burst-forming unit-erythroid
bg: *beige*
BM: bone marrow
BSA: bovine serum albumin
BZ: border zone
CABG: coronary artery bypass graft
CASO: Casein peptone Soybean flour peptone
CD: cluster of differentiation
CFU: colony-forming unit
CFU-E: CFU-erythroid
CFU-EC: CFU-endothelial cell
CFU-GEMM: CFU-granulocyte, erythrocyte, macrophage, megakaryocyte
CFU-GM: CFU-granulocyte, macrophage
CFU-H: hematopoietic CFU
CP: cell product
DAPI: 4′,6-diamidino-2-phenylindole
EDTA: ethylenediaminetetraacetic acid
EDV: end-diastolic volume
EF: ejection fraction
EGM: Endothelial Cell Growth Medium
EMA: European Medicines Agency
ESV: end-systolic volume
FACS: fluorescence-activated cell sorting
FMO: fluorescence minus one
GLP: Good Laboratory Practice
GMP: Good Manufacturing Practice
HPF: high-power field
HSA: human serum albumin
ILAB: Institute for Clinical Chemistry and Laboratory Medicine
IMIKRO: Institute for Medical Microbiology, Virology and Hygiene
IS: infarcted scar
ISHAGE: International Society of Hematotherapy and Graft Engineering
LAD: left anterior descending coronary artery
LAF: laminar air flow
LALLF M-V: Landwirtschaft, Lebensmittelsicherheit und Fischerei Mecklenburg-Vorpommern
LV: left ventricle
LVEF: left ventricular ejection fraction
MACS: magnetic cell sorting
MI: myocardial infarction
MIC: MI control group
NTCB: non-target cell bag
OECD: Organisation for Economic Co-operation and Development
PBS: phosphate-buffered saline
PFA: paraformaldehyde
PQR: product quality review
Prodigy: CliniMACS Prodigy® System
PV: pressure-volume
QC: quality control
RA: remote area
RT: room temperature
SCID: severe combined immunodeficient
SEM: standard error of the mean
TS: tubing set
vWF: von Willebrand factor
WB: waste bag
