# Supplementary figures and images for: GMP-conformant on-site manufacturing of a CD133+ stem cell product for cardiovascular regeneration
Source: Stem Cell Res Ther. 2017 Feb 10;8:33. doi: 10.1186/s13287-016-0467-0 (PMC5303262; doi:10.1186/s13287-016-0467-0)

**a**

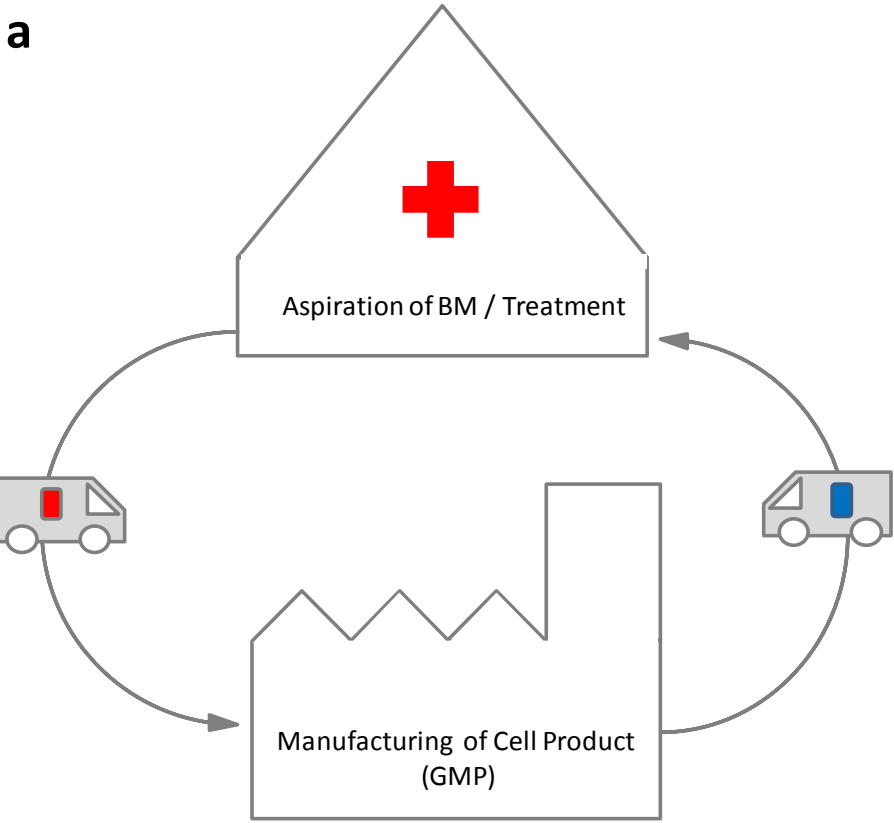

**b**

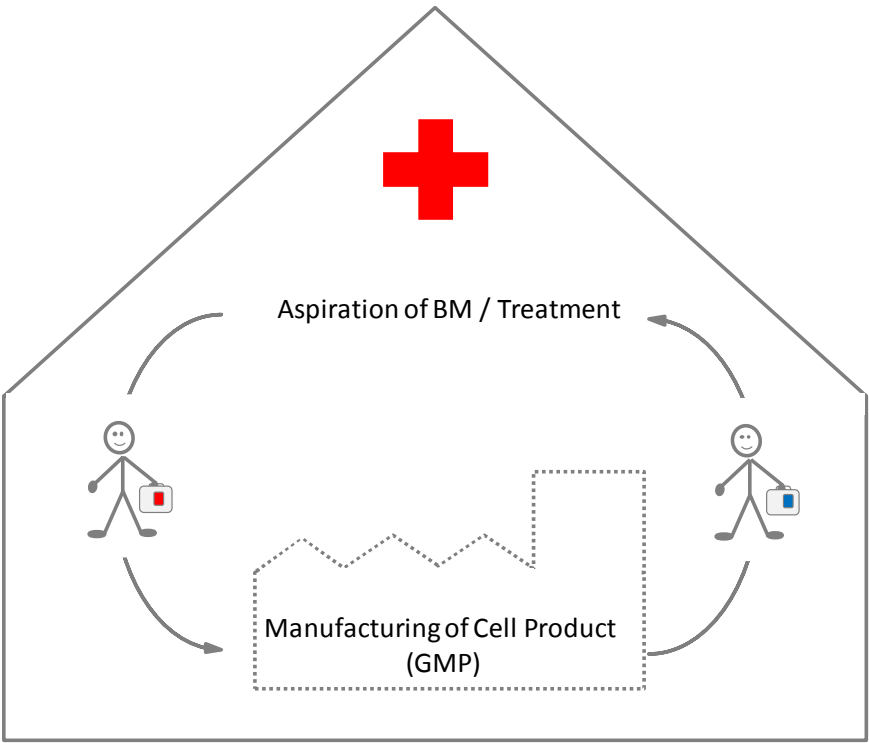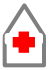

hospital

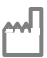

external  
manufacturing site

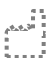

internal  
manufacturing site

Supplement: Additional file 1: Figure S1. — Schematic representation of two different logistical procedures used for the manufacturing of stem cell products in cardiovascular regenerative medicine. Manual/semi-automatic systems require centralized GMP-compliant manufacturing by an external service provider (a). Entirely closed systems such as the automatic CliniMACS Prodigy® enable decentralized GMP-compliant manufacturing by an in-house facility (b). a Centralized GMP-compliant manufacturing by an external service provider: few specialized contractors are available for a distinct cell product because standardized high-level clean rooms (Class A in B) and sophisticated training are required for the manual or semi-automatic manufacturing. This results in higher costs of the therapy due to an increased logistical effort (e.g., long-distance transportation) and longer hospitalization of the patients as well as a possible loss of cell product quality (e.g., CliniMACS® Plus). b Decentralized GMP-compliant manufacturing by an in-house facility: cell products intended for clinical application can be manufactured on-site owing to reduced clean room requirements (Class A in D) and easier standardization of processes with minimized inter-individual variability due to automatic manufacturing. Lower logistical effort (in-house transport) and shorter hospitalization enables minimized costs plus a better and stable quality of the cell product (e.g., CliniMACS Prodigy®). (PDF 109 kb) [file 13287_2016_467_MOESM1_ESM.pdf]

# CliniMACS Prodigy® BM-133 system

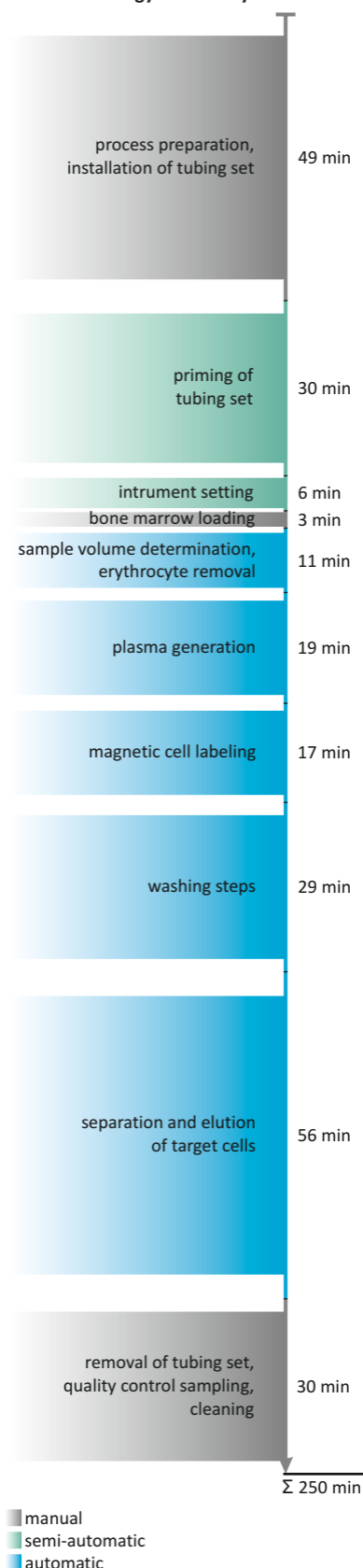

Supplement: Additional file 4: Figure S2. — Schematic schedule of the CD133+ cell isolation process using the CliniMACS Prodigy® BM-133 system. Gray: manual steps (two operators); green: semi-automatic steps (one operator, dual control); blue: automatic steps. (PDF 1368 kb) [file 13287_2016_467_MOESM4_ESM.pdf]

## CD34/CD133 expression

## size/granularity

**a**

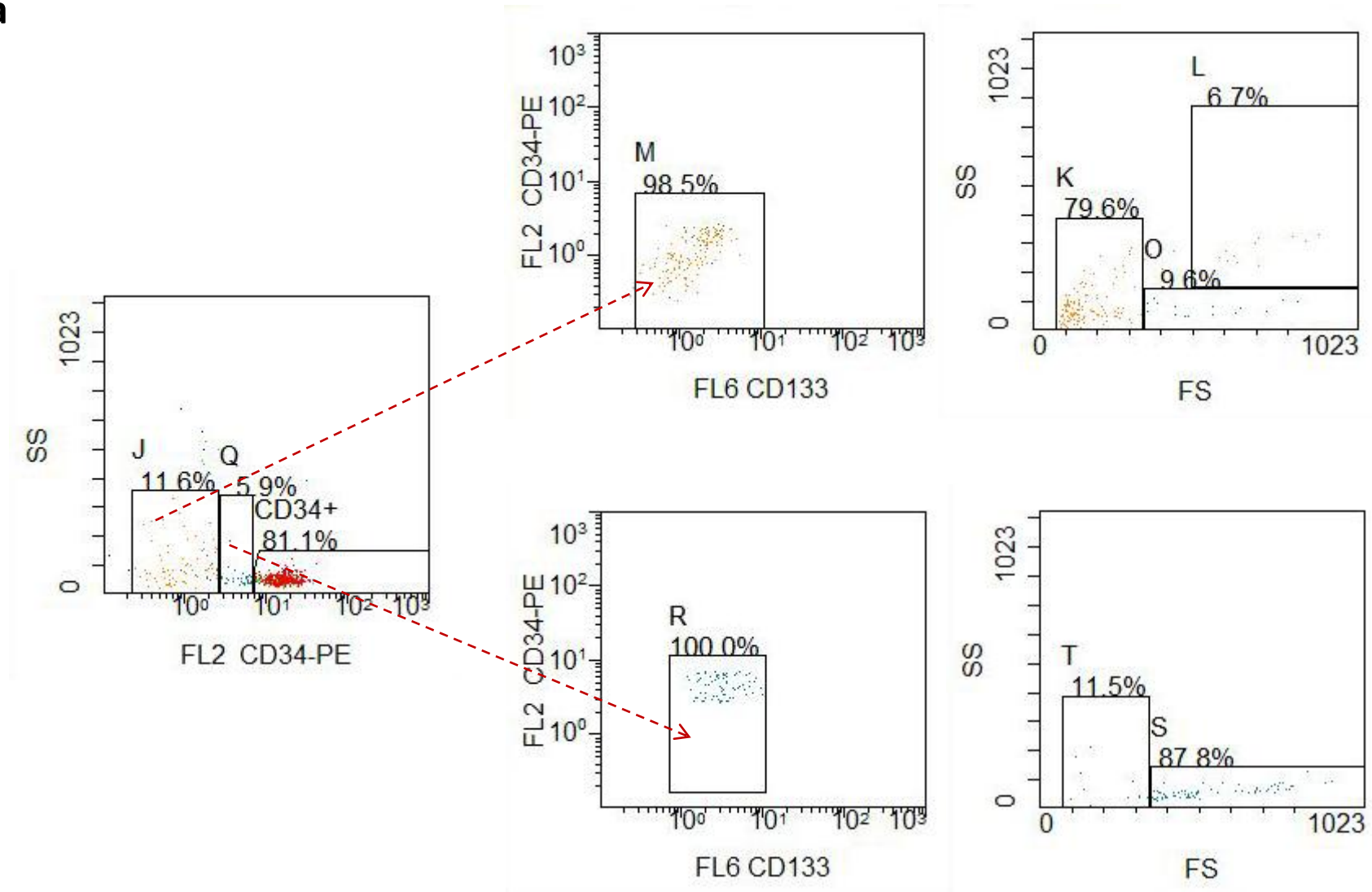

**b**

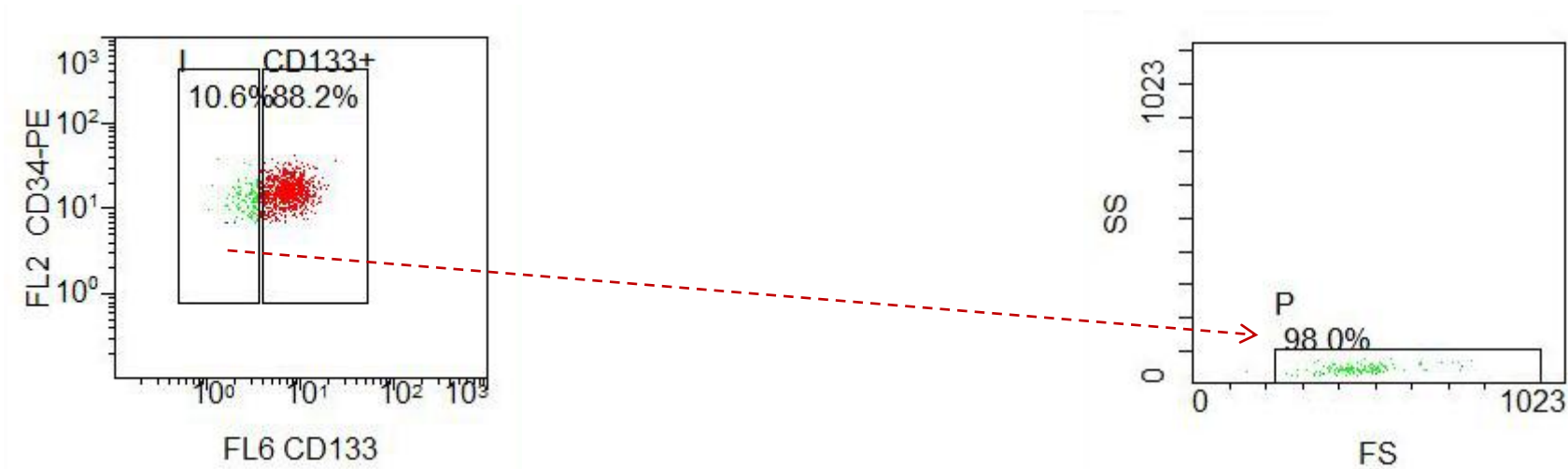

Supplement: Additional file 7: Figure S6. — Phenotypic and morphologic characterization of non-target cell populations presented in the automatically generated cell product (CP). Non-target cell populations were subdivided into three fractions: CD45+/CD34− (region J) and CD45+/CD34low (region Q) (a); CD45+/CD34+/CD133− (region I) (b). CD45+/CD34-/low non-target cells were further analyzed concerning their CD133 expression (regions M and R, respectively). Size and granularity of all three fractions were evaluated: region K and T: smaller than target cell population; region O, S and P: same size/granularity than target cell population; region L: higher granularity than target cell population. (PDF 130 kb) [file 13287_2016_467_MOESM7_ESM.pdf]

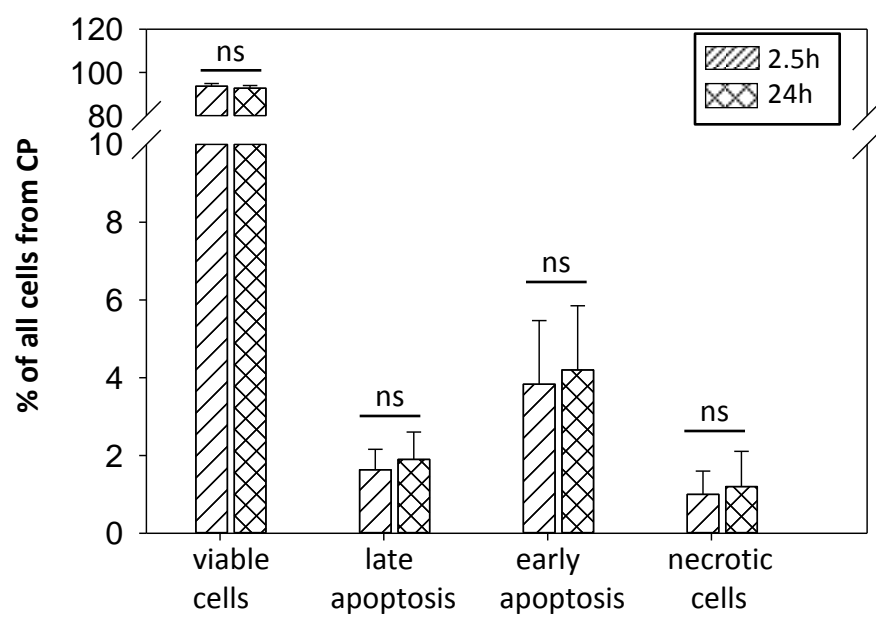

Supplement: Additional file 9: Figure S7. — Determination of apoptotic and necrotic cells in the automatically generated cell product (CP) after storage. The percentage of viable, early-stage apoptotic, late-stage apoptotic and necrotic cells was analyzed by flow cytometric measurements using Annexin V Apoptosis Detection Kit. Samples of CP were taken at the respective storage time. All data are presented as a mean ± SEM. n = 3. *p ≤ 0.05; **p ≤ 0.01; ***p ≤ 0.001. (PDF 108 kb) [file 13287_2016_467_MOESM9_ESM.pdf]

## acLDL

manual

automatic

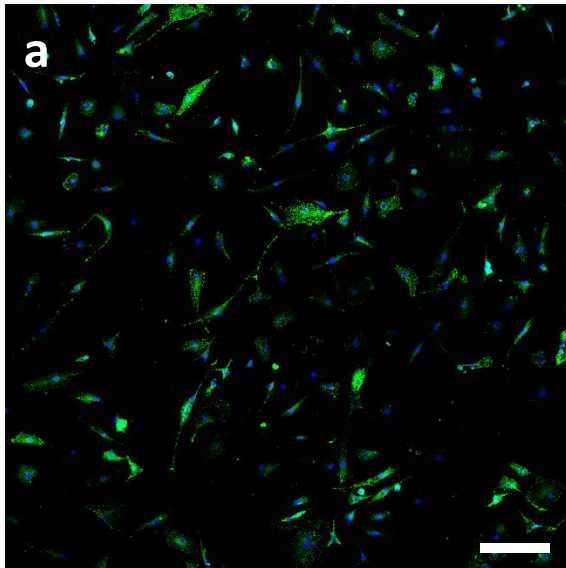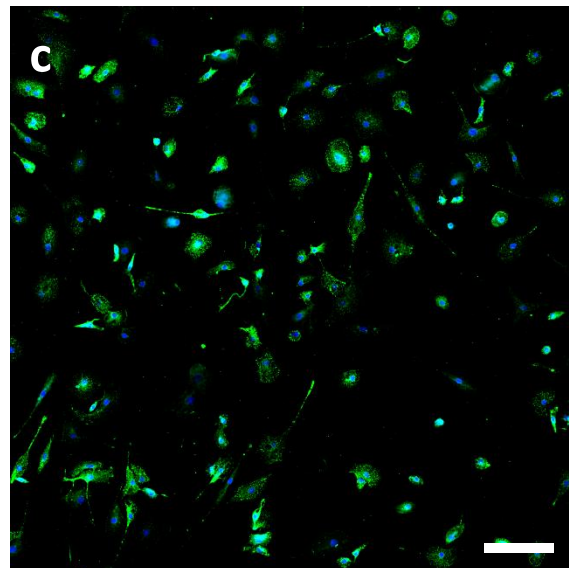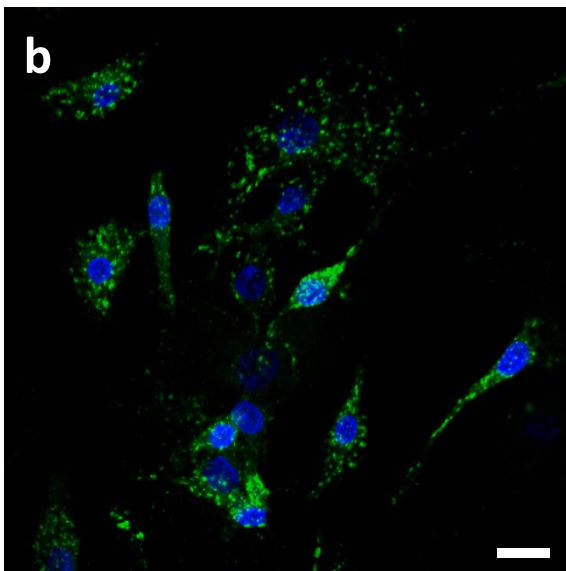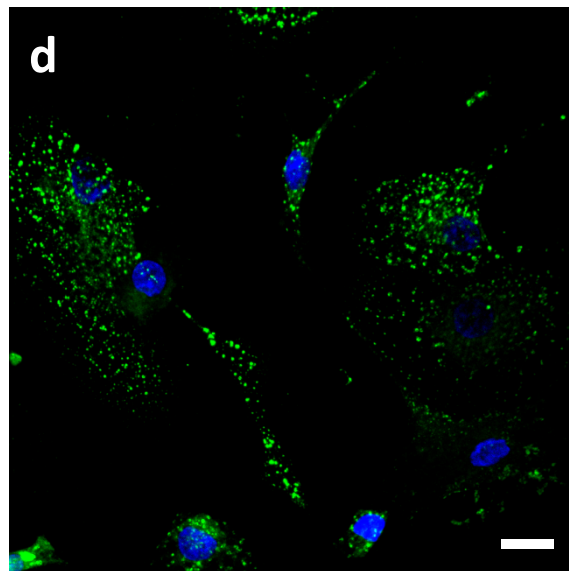

Supplement: Additional file 10: Figure S3. — Uptake of acetylated low-density lipoprotein (acLDL) of colony-forming unit endothelial cell (CFU-EC) assay-derived cells. CFU-EC assay was performed and after 29 days incubation the uptake of acLDL (green) was examined by immunostaining in non-adherent cells. Nuclei were stained with Hoechst dye (blue). Pictures were taken using ELYRA PS.1 LSM 780 microscope (Carl Zeiss GmbH). Images of manually (a and b) and automatically (c and d) isolated cells. Scale bars: 100 μm (a and c) and 20 μm (b and d). (PDF 341 kb) [file 13287_2016_467_MOESM10_ESM.pdf]

**vWF**

manual

automatic

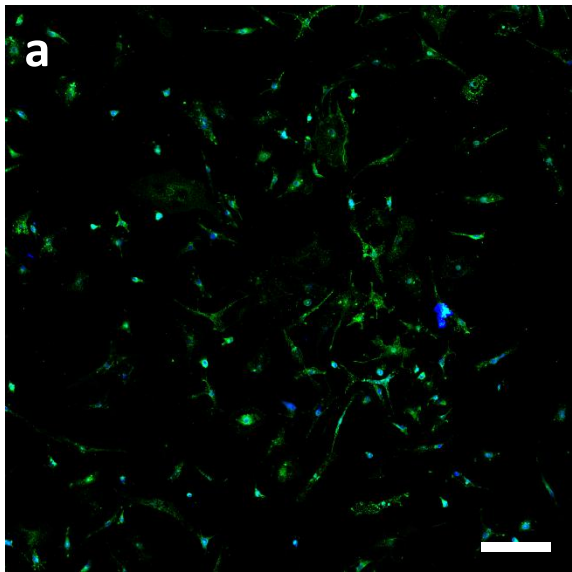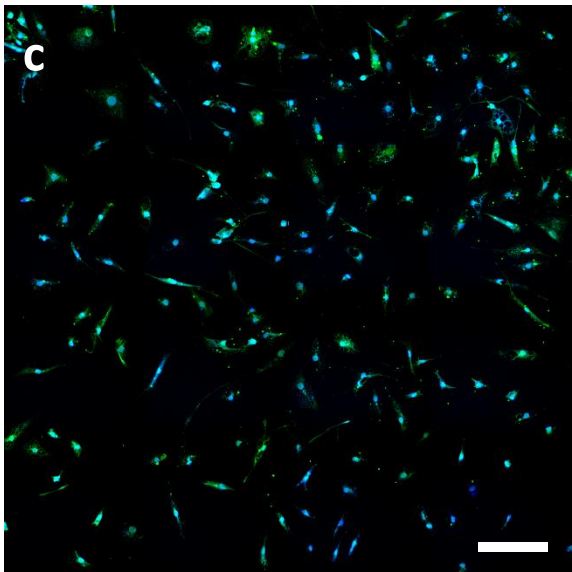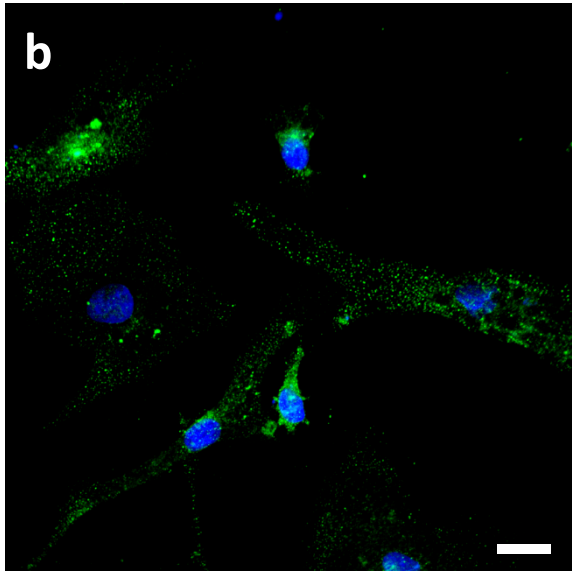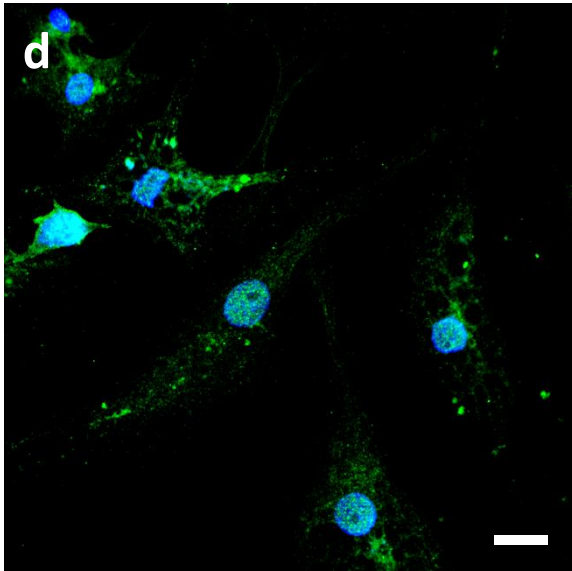

Supplement: Additional file 11: Figure S4. — Von Willebrand factor (vWF) expression of colony-forming unit endothelial cell (CFU-EC) assay-derived cells. CFU-EC assay was performed and after 29 days incubation the vWF expression (green) was examined by immunostaining in non-adherent cells. Nuclei were stained with Hoechst dye (blue). Pictures were taken using ELYRA PS.1 LSM 780 microscope (Carl Zeiss GmbH). Images of manually (a and b) and automatically (c and d) isolated cells. Scale bars: 100 μm (a and c) and 20 μm (b and d). (PDF 295 kb) [file 13287_2016_467_MOESM11_ESM.pdf]
