# Supplementary material for: GMP-conformant on-site manufacturing of a CD133+ stem cell product for cardiovascular regeneration
Source: Stem Cell Res Ther. 2017 Feb 10;8:33. doi: 10.1186/s13287-016-0467-0 (PMC5303262; doi:10.1186/s13287-016-0467-0)
Supplement: Additional file 2: Table S1. — Overview of data obtained from CliniMACS® Prodigy BM-133 System validation process (a) and product quality review (PQR) (b). Volumes of samples were ascertained by visual control (bone marrow (BM)) or by weighing (cell product (CP), non-target cell bag (NTCB), waste bag (WB)). Total numbers of viable CD45+ and CD133+ cells were calculated by multiplying absolute cell numbers per volume unit with corresponding volumes. The frequencies of viable CD133+ cells were measured by flow cytometry in accordance with ISHAGE guidelines. (DOC 42 kb) [file 13287_2016_467_MOESM2_ESM.doc]

**a**

| **Validation No.** | **Non-diluted filtered BM** | | | | **CP** | | | |
| --- | --- | --- | --- | --- | --- | --- | --- | --- |
|  | **Volume [ml]** | **Total no. of viable CD45^+^ cells (×10^6^)** | **Total no. of viable CD133^+^ cells (×10^6^)** | **Frequency of viable CD133^+^ cells [%]** | **Volume [ml]** | **Total no. of viable CD45^+^ cells (×10^6^)** | **Total no. of viable CD133^+^ cells (×10^6^)** | **Frequency of viable CD133^+^ cells [%]** |
| **1** | 65 | 1533.03 | 8.22 | 0.54 | 9.26 | 2.06 | 1.85 | 89.86 |
| **2** | 59 | 435.92 | 1.80 | 0.41 | 6.69 | 1.03 | 0.37 | 36.25 |
| **3** | 63 | 611.51 | 1.95 | 0.32 | 8.53 | 2.51 | 0.97 | 38.61 |
| **4** | 60 | 319.68 | 0.66 | 0.21 | 7.67 | 0.40 | 0.28 | 71.84 |
| **5** | 47 | 224.68 | 0.63 | 0.28 | 8.49 | 0.28 | 0.21 | 74.24 |
| **6** | 61 | 852.44 | 3.20 | 0.38 | 8.15 | 2.14 | 1.57 | 73.38 |
| **Mean** | **59.17** | **662.88** | **2.74** | **0.36** | **8.13** | **1.40** | **0.88** | **64.03** |
| **SEM** | 2.59 | 196.39 | 1.16 | 0.05 | 0.36 | 0.39 | 0.29 | 8.83 |

**b**

| **Validation No.** | **NTCB** | | | | **WB** | | | |
| --- | --- | --- | --- | --- | --- | --- | --- | --- |
|  | **Volume [ml]** | **Total no. of viable CD45^+^ cells (×10^6^)** | **Total no. of viable CD133^+^ cells (×10^6^)** | **Frequency of viable CD133^+^ cells [%]** | **Volume [ml]** | **Total no. of viable CD45^+^ cells (×10^6^)** | **Total no. of viable CD133^+^ cells (×10^6^)** | **Frequency of viable CD133^+^ cells [%]** |
| **1** | 236.66 | 1199.63 | 4.50 | 0.37 | 1686.94 | 8.43 | 0.00 | 0.00 |
| **4** | 237.81 | 246.85 | 0.24 | 0.10 | 1686.27 | 5.06 | 0.00 | 0.00 |
| **6** | 238.61 | 631.60 | 1.19 | 0.19 | 1696.71 | 10.18 | 0.00 | 0.00 |
| **Mean** | **237.69** | **692.69** | **1.98** | **0.22** | **1689.97** | **7.89** | **0.00** | **0.00** |
| **SEM** | 0.57 | 276.74 | 1.29 | 0.08 | 3.37 | 1.50 | - | - |
