# Supplementary material for: GMP-conformant on-site manufacturing of a CD133+ stem cell product for cardiovascular regeneration
Source: Stem Cell Res Ther. 2017 Feb 10;8:33. doi: 10.1186/s13287-016-0467-0 (PMC5303262; doi:10.1186/s13287-016-0467-0)
Supplement: Additional file 3: Table S2. — Overview of data obtained from manual cell isolation. Volumes of bone marrow (BM) samples were ascertained by visual control. Total numbers of viable CD133+ cells were determined using Neubauer hemocytometer. The frequencies of viable CD133+ cells were measured by flow cytometry in accordance with ISHAGE guidelines. (DOC 36 kb) [file 13287_2016_467_MOESM3_ESM.doc]

| **Manual isolation No.** | **BM** | | **CD133^+^** | |
| --- | --- | --- | --- | --- |
|  | **Volume [ml]** | **Frequency of viable CD133^+^ cells**  **[%]** | **Total no. of viable CD133^+^ cells (×10^6^)** | **Frequency of viable CD133^+^ cells [%]** |
| 1 | 60 | 0.34 | 0.23 | 83.48 |
| 2 | 55 | 0.42 | 0.29 | 82.75 |
| 3 | 60 | 0.73 | 0.60 | 89.72 |
| 4 | 54 | 0.30 | 0.76 | 86.81 |
| 5 | 50 | 0.35 | 0.20 | 90.99 |
| 6 | 60 | 0.76 | 1.17 | 95.35 |
| **Mean** | **56.50** | **0.48** | **0.54** | **88.18** |
| SEM | 1.71 | 0.08 | 0.16 | 1.96 |
