# Supplementary material for: GMP-conformant on-site manufacturing of a CD133+ stem cell product for cardiovascular regeneration
Source: Stem Cell Res Ther. 2017 Feb 10;8:33. doi: 10.1186/s13287-016-0467-0 (PMC5303262; doi:10.1186/s13287-016-0467-0)
Supplement: Additional file 5: Table S3. — List of antibodies used for flow cytometry-based quality control of manually isolated CD133+ stem cells and for the characterization of stemness marker expression in the automatically generated cell product. (DOC 33 kb) [file 13287_2016_467_MOESM5_ESM.doc]

| **Name antibody /isotype** | **Fluorochrome- conjugate** | **Clone** | **Company** |
| --- | --- | --- | --- |
| CD133 | phycoerythrin, PE | 293C2 | Miltenyi Biotec |
| Mouse isotype IgG2a | PE | - | Miltenyi Biotec |
| CD34 | fluorescein, FITC | AC136 | Miltenyi Biotec |
| CD45 | allophycocyanine tandem, APC-H7 | 2D1 | Becton Dickinson (BD) |
| **Name antibody ( Stemness marker)** | | | |
| CD34 | FITC | AC136 | Miltenyi Biotec |
| CD133 | PE | 293C2 | Miltenyi Biotec |
| CD184 | phycoerythrin-cyanine tandem, PE-Cy5 | 12G5 | BD |
| CD117 | PE-Cy7 | 104D2 | BD |
| CD309 | APC | ES8-20E6 | Miltenyi Biotec |
| CD14 | Pacific-blue, V450 | MΦP9 | BD |
| CD45 | AmCyan,V500 | HI30 | BD |
