# Supplementary material for: GMP-conformant on-site manufacturing of a CD133+ stem cell product for cardiovascular regeneration
Source: Stem Cell Res Ther. 2017 Feb 10;8:33. doi: 10.1186/s13287-016-0467-0 (PMC5303262; doi:10.1186/s13287-016-0467-0)
Supplement: Additional file 6: Figure S5. — Representative ISHAGE-based gating strategy used for the quality control of the automatically generated cell product (CP). Debris was excluded from CD45+ cells (a). CD34+ cells were selected from viable CD45+ cells (b). Events with high expression of the CD45 marker were excluded from viable CD45+/CD34+cells (c). FSC/SSC backgate was employed to select viable CD45+/CD34+ hematopoietic progenitor cells (HPCs) with blast morphology (d). Viable CD45+/CD34+/CD133+ cells were selected (e). Events with high expression of the CD45 marker were excluded from viable CD45+/CD34+/CD133+ cells (f). FSC/SSC backgate was employed to select viable CD45+/CD34+/CD133+ HPCs with blast morphology (g). A control gate (‘Ly’, lymphocytes) was used during exclusion of mature CD45+ HSCs. Red: target cell population. Gray: dead cells. (PDF 374 kb) [file 13287_2016_467_MOESM6_ESM.pdf]

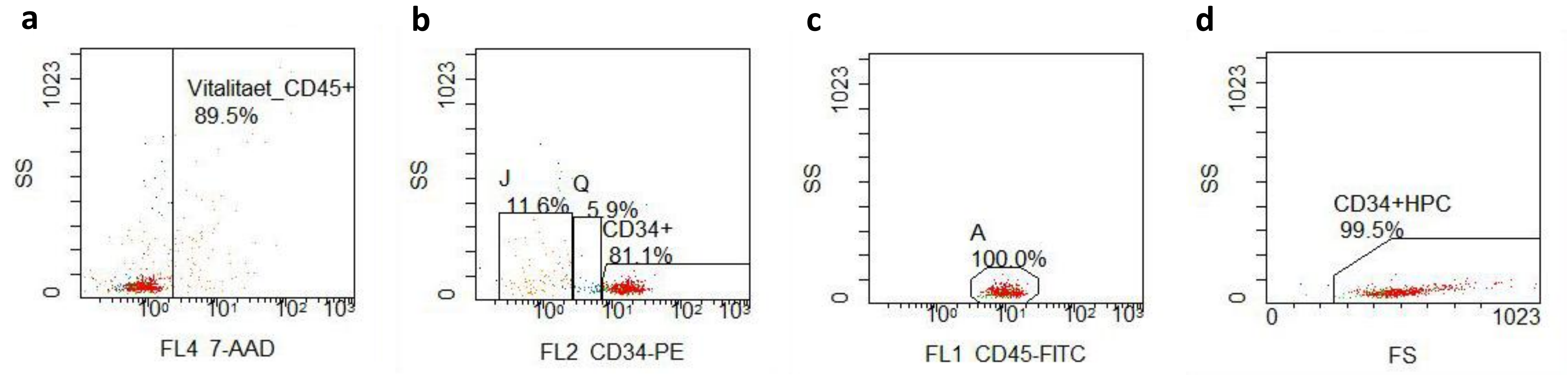

a

Region	Cells/µL	Number	%Gated
ALL	304	2637	100.00
Vitalitaet_CD45+	272	2360	89.50

b

Region	Cells/µL	Number	%Gated
ALL	272	2360	100.00
CD34+	220	1913	81.06
J	32	274	11.61
Q	16	139	5.89

c

Region	Cells/µL	Number	%Gated
ALL	220	1913	100.00
A	220	1913	100.00

d

Region	Cells/µL	Number	%Gated
ALL	220	1913	100.00
CD34+HPC	219	1903	99.48

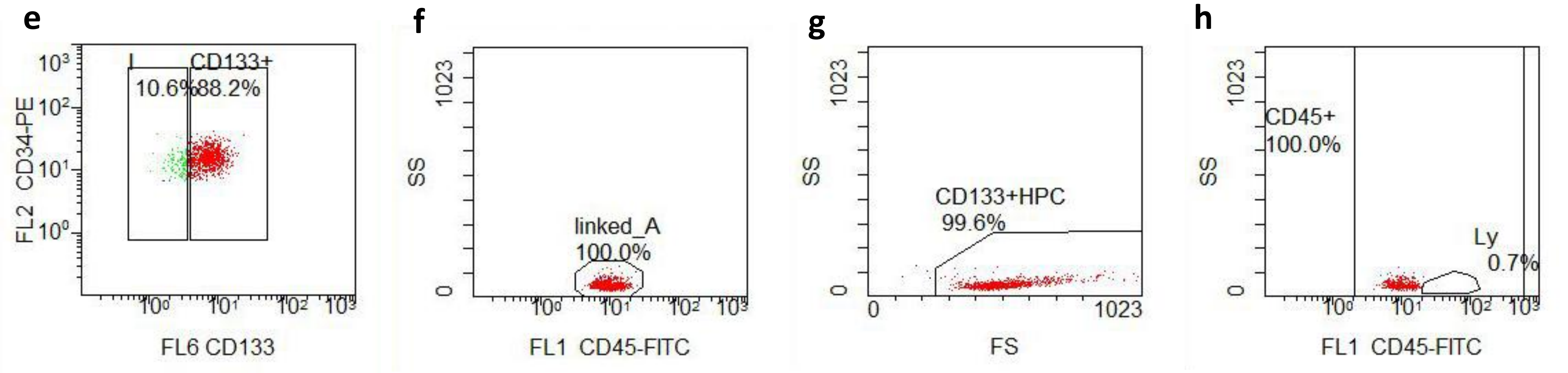

e

Region	Cells/µL	Number	%Gated
ALL	220	1913	100.00
CD133+	195	1693	88.50
I	23	198	10.35

f

Region	Cells/µL	Number	%Gated
ALL	195	1693	100.00
linked_A	195	1693	100.00

g

Region	Cells/µL	Number	%Gated
ALL	195	1693	100.00
CD133+HPC	194	1686	99.59

h

Region	Cells/µL	Number	%Gated
ALL	200	1740	100.00
CD45+	200	1740	100.00
Ly	1	12	0.69
