# Supplementary material for: GMP-conformant on-site manufacturing of a CD133+ stem cell product for cardiovascular regeneration
Source: Stem Cell Res Ther. 2017 Feb 10;8:33. doi: 10.1186/s13287-016-0467-0 (PMC5303262; doi:10.1186/s13287-016-0467-0)
Supplement: Additional file 8: Table S4. — Stemness marker expression of target and non-target cell fractions presented in the automatically generated cell product (CP). For phenotype characterization the expression of stemness markers (CD117, CD184, CD309, CD14) was evaluated in target cells (CD45+CD34+CD133+) (a) and non-target cells (CD45+CD34−CD133−, CD45+CD34lowCD133−, CD45+CD34+CD133−) (b) using flow cytometry. The respective analysis was made in accordance with ISHAGE guidelines. Data are presented as a mean ± SEM, n = 9. (DOC 37 kb) [file 13287_2016_467_MOESM8_ESM.doc]

**a**

| **Target cell fraction** | **% of CD45^+^/CD34^+^/CD133^+^** | | | |
| --- | --- | --- | --- | --- |
| **Stemness marker** | CD117^+^ | CD184^+^ (CXCR4) | CD309^+^  (KDR) | CD14^+^ |
|
| **Mean** | 71.81 | 8.24 | 1.83 | 0.10 |
| **SEM** | 4.35 | 1.59 | 0.36 | 0.04 |

**b**

| **Non-target cell fraction** | **% of CD45^+^/CD34^-^/CD133^-^**  **(region J)** | | | | **% of CD45^+^/CD34^low^/CD133^-^**  **(region Q)** | | | | **% of CD45^+^/CD34^+^/CD133^-^**  **(region I)** | | | |
| --- | --- | --- | --- | --- | --- | --- | --- | --- | --- | --- | --- | --- |
| **Stemness marker** | CD117^+^ | CD184^+^ (CXCR4) | CD309^+^  (KDR) | CD14^+^ | CD117^+^ | CD184^+^ (CXCR4) | CD309^+^  (KDR) | CD14^+^ | CD117^+^ | CD184^+^ (CXCR4) | CD309^+^  (KDR) | CD14^+^ |
|
| **Mean** | 10.02 | 34.69 | 14.00 | 11.03 | 31.47 | 22.74 | 2.98 | 1.03 | 36.69 | 9.62 | 1.61 | 0.22 |
| **SEM** | 3.09 | 7.59 | 7.44 | 2.78 | 5.63 | 3.14 | 0.55 | 0.30 | 5.52 | 1.34 | 0.40 | 0.12 |
